# Editorial: Sedentary behaviour and cardiometabolic health

**DOI:** 10.3389/fcvm.2024.1498410

**Published:** 2024-10-16

**Authors:** Liane Beretta De Azevedo, Gabriel Grizzo Cucato, Bente Morseth

**Affiliations:** ^1^ College of Health, Wellbeing and Life Sciences, Sheffield Hallam University, Sheffield, United Kingdom; ^2^ Department of Sport, Exercise and Rehabilitation, Northumbria University, London, United Kingdom; ^3^ School of Sport Sciences, UiT The Arctic University of Norway, Tromsø, Norway

**Keywords:** sedentary behavior, cardiovascular, cardiometabolic, public health, behaviour change

**Editorial on the Research Topic**
Sedentary behaviour and cardiometabolic health

Sedentary behaviour is a growing public health issue, with the latest World Health Organisation (WHO) physical activity guidelines now including recommendations regarding sedentary behaviour due to the increasing body of evidence showing the harmful effects of sedentary behaviour on health outcomes (1). In an overview of 18 systematic reviews, Saunders et al., (2) concluded that sedentary behaviour was associated with depression, poor cognitive function and low physical-related quality of life. The review of reviews also highlighted that breaking up sedentary behaviour may improve body composition and markers of cardiometabolic risks. It has been reported that Americans spend 55% of their waking time (7.7 h/day) on sedentary behaviour (3). A more recent epidemiological study in 28 European countries showed an increase in the prevalence of sedentary behaviour (defined by sitting for longer than 4 h 40 min a day) for both men and women between 2002 and 2017, with a higher prevalence in men (4).

Although sedentary behaviour is on the rise, cardiovascular disease and mortality incidence have been declining since 1990, especially in developed countries compared to developing counterparts. This decline is associated with better access to medication and a healthy lifestyle (5). However, cardiovascular disease remains the most prevalent non-communicable disease worldwide. It is estimated that CVD causes 17.9 million deaths per year, representing 32% of global deaths (6).

This Research Topic contains studies that discuss the prevalence of sedentary behaviour and its potential mechanisms. It also explores the impact of breaking up sedentary time on cardiometabolic markers. Lastly, it examines men's perception of the threat of cardiovascular diseases and the effectiveness of behaviour change.

The study by Liang et al., analysed a sample of 5,610 respondents with cardiovascular disease from the US National Health and Nutrition Examination Survey (NHANES). In contrast to the study by Lopes-Valenciano (4), which found an increase in prevalence in European countries, Liang et al. found that the prevalence of sedentary behaviour, physical activity levels, and smoking remained stable between 1990 and 2000 and 2017–2018 in the USA. However, the prevalence of poor diet decreased. Surprisingly, there was a significant increase in obesity, which is difficult to explain considering the reduction in poor diet and maintenance of physical activity and sedentary behaviour. The authors, however, performed a sub-analysis which showed that the group with low education and income were at a higher risk of being exposed to unhealthy lifestyle factors such as sedentary behaviour compared to those at the other end of the spectrum (i.e., high education and income levels), justifying the needs for targeted interventions for this population.

Two studies in this Research Topic explored the mechanisms of the association between sedentary behaviour and cardiometabolic markers. It is known that high levels of leptin are related to obesity and cardiovascular disease (7). In a cross-sectional study, Shih et al. found a positive association between the high leptin group and sedentary behaviour, after adjusting to other behaviours and metabolic markers. The association between sedentary time and leptin levels was observed in individuals with both high BMI and normal BMI, suggesting that BMI was not the sole reason for the increase in leptin levels. The authors suggested that other factors, such as elevated sympathetic tone, may also play a role (8). The authors concluded that sedentary behaviour independently contributes to increased leptin levels and highlighted the importance of reducing sedentary behaviour to lower leptin levels and prevent cardiovascular diseases.

On the other hand, Vandercappellen et al. investigated the association between sedentary behaviour and physical activity levels with biomarkers of cardiac injury in a large population (The Maastricth study). After controlling for demographic and other lifestyle risk factors, they did not find an association between physical activity, sedentary time, and cardiac troponins, contradicting previous findings from the literature (9). However, they found that vigorous and moderate to vigorous physical activity were associated with lower levels of NT-pro BNP (a biomarker often associated with cardiac stress and heart failure), suggesting that high-intensity physical activity may benefit cardiovascular function.

The literature has highlighted the adverse effects of continuous and prolonged sedentary behaviour, which is still a prevalent issue (10). Silva et al. assessed sedentary behaviour using accelerometers in a sample of 184 adults aged 18–59 years. The authors observed that younger adults spent more time in sedentary bouts (uninterrupted sedentary behaviour) than middle-aged adults. However, time on sedentary breaks (interruption of sedentary bouts with physical activity) was similar. The authors attribute the increase in the use of interactive electronic devices by the younger population as a potential reason for the prolonged time in sedentary bouts. However, while other studies have found an association between cardiovascular markers and sedentary behaviour patterns (11), only age showed a significant correlate in this study.

Domosławska-Żylińska et al. conducted a survey of 1,000 Polish men to assess their perceived susceptibility to cardiovascular disease and the efficacy of preventive behaviours such as physical activity. Despite 54.2% recognising the severity of cardiovascular disease, only 15.1% perceived themselves as being at risk. Most individuals expressed confidence in the effectiveness of preventive measures, including regular physical activity, a healthy diet, and medication adherence. However, the responsive (high threat, high efficacy) and proactive (low threat, high efficacy) groups reported higher engagement in preventive behaviours compared to the indifferent group (low threat, low efficacy). The study concluded that increasing self-efficacy is essential to improving preventive behaviour uptake in men.

The articles in this Research Topic offer additional evidence regarding the connection between sedentary behaviour and cardiovascular health. This includes insights into the mechanisms of the association, prevalence and patterns and perceived risks of cardiovascular disease, and the efficacy of modifying preventative behaviours. While more research is needed to guide public health guidelines on the relationship between sedentary behaviour and cardiometabolic outcomes, we hope that this issue has contributed to advancing our knowledge in this area.
